# Loss of mDia1 and Fhod1 impacts platelet formation but not platelet function

**DOI:** 10.1080/09537104.2020.1822522

**Published:** 2020-09-27

**Authors:** Malou Zuidscherwoude, Elizabeth J. Haining, Victoria A. Simms, Stephanie Watson, Beata Grygielska, Alex T. Hardy, Andrea Bacon, Stephen P. Watson, Steven G. Thomas

**Affiliations:** 1 Institute of Cardiovascular Sciences, College of Medical and Dental Sciences, University of Birmingham, Birmingham, UK; 2 Centre of Membrane Proteins and Receptors (COMPARE), University of Birmingham and University of Nottingham, Midlands, UK; 3 Genome Editing Facility, Technology Hub, College of Medical and Dental Sciences, University of Birmingham, Birmingham, UK

**Keywords:** Actin, cytoskeleton, Fhod1, formin, macrothrombocytopenia, mDia1, microtubules

## Abstract

An organized and dynamic cytoskeleton is required for platelet formation and function. Formins are a large family of actin regulatory proteins which are also able to regulate microtubule dynamics. There are four formin family members expressed in human and mouse megakaryocytes and platelets. We have previously shown that the actin polymerization activity of formin proteins is required for cytoskeletal dynamics and platelet spreading using a small molecule inhibitor. In the current study, we analyze transgenic mouse models deficient in two of these proteins, mDia1 and Fhod1, along with a model lacking both proteins. We demonstrate that double knockout mice display macrothrombocytopenia which is due to aberrant megakaryocyte function and a small decrease in platelet lifespan. Platelet function is unaffected by the loss of these proteins. This data indicates a critical role for formins in platelet and megakaryocyte function.

## Introduction

Megakaryocytes and platelets require a dynamic and properly regulated actin cytoskeleton for megakaryocyte development, platelet formation, and platelet function. In megakaryocytes, cytoplasmic maturation via endomitosis and the formation of the demarcation membrane system are both dependent on the cytoskeleton [1,2] as are proplatelet extension, branching, and platelet release [3–5]. The importance of a dynamic cytoskeleton in platelet formation is evidenced by the identification of mutations in multiple cytoskeletal regulatory proteins which result in thrombocytopenia [6,7]. Likewise, the role of the actin cytoskeleton in the maintenance of platelet shape on its journey through the circulation and upon platelet activation is well established. The cytoskeleton is critical for shape change, spreading, and clot retraction [8,9], and is regulated by a plethora of actin binding and regulatory proteins, and disruption to these often results in defects of platelet function (e.g [6,10,11]).

Formins are a class of actin-binding proteins known as nucleation-promoting factors, although they have been shown to have functions beyond simple nucleation. Members of the formin protein family have the ability to both nucleate and accelerate the elongation of linear actin fibers and are also involved in microtubule dynamics and organization [12]. Formins are increasingly recognized as regulators of cross-talk between the actin and microtubule cytoskeletons and they can therefore be expected to play a role in multiple aspects of platelet formation and function [13]. Indeed, a gain of function variant of formin mDia1 (DIAPH1) is linked to macrothrombocytopenia in humans [14,15]. Of the 15 mammalian formins, only 4 are expressed in platelets and megakaryocytes, namely mDia1, Fhod1, Daam1, and Inf2 which contribute 24.5%, 36.7%, 14.5%, and 24.3%, respectively, of the total formin protein expressed in mouse platelets [13,16]. We have previously shown that platelets from an mDia1 knockout mouse were functionally normal and hypothesize that this was due to redundant function with other platelet/megakaryocyte expressed formin family members [16]. In support of this, we have recently reported that blocking formin FH2 domains using a small molecule inhibitor (SMIFH2) completely abolishes platelet spreading and disrupts microtubule dynamics [17], highlighting the role of formins in regulating both actin and microtubule platelet cytoskeletons.

In the current study we generated a Fhod1-deficient transgenic mouse model, and subsequently crossed this with the mDia1-deficient mouse to generate a double mDia1/Fhod1-deficient mouse. This allowed us to assess the effect of loss of these formin family members on platelet formation and function. Here we show that loss of both mDia1 and Fhod1 results in macrothrombocytopenia due to defective platelet production. However, platelet function is unaffected by the loss of these two proteins.

## Methods

### Generation of mDia1 and Fhod1 Knockout Mouse Models

Constitutive Fhod1 knockout mice were generated by injection of gene-trapped embryonic stem cells into blastocyst stage embryos derived from super-ovulated female mice using methods as previously described [18]. Gene trapped ES cells for Fhod1 (IST13553G8) were obtained from the Texas Institute for Genomic Medicine (https://www.tigm.org). Details of the gene trap vector and genotyping of the mice are given in supplementary figures (Supp. & 2). mDia1 knockout mice were provided by Prof. Art Alberts (generated as previously described [19]). All mice were on a C57BLK6 background. Animals were maintained using housing and husbandry in accordance with local and national legal regulations and all work was performed under appropriate Home Office licenses on mice between 8 and 24 weeks of age and littermates were used as wild type controls. Calculations of Mendelian inheritance patterns for mDia1 and Fhod1 transgenic mice were carried out as per [20].

### Platelet Preparation

Blood was drawn from the vena cava of CO_2_ narcosed mice directly into 100 μL acid/citrate/dextrose (ACD). The number and size of platelets in whole blood was determined using an ABX Micros 60 hematological analyzer (ABX Diagnostics, Montpelier, France). PRP was obtained by centrifugation at 200x g for 6 min. Washed platelets were prepared via centrifugation of PRP at 1000x g in the presence of 0.1 µg ml^−1^ prostacyclin (Caymen Chemicals) for 6 min. The platelet pellet was resuspended in modified Tyrodes buffer (134 mM NaCl, 0.34 mM Na_2_HPO_4_, 2.9 mM KCl, 12 mM NaHCO_3_, 20 mM HEPES, 5 mM glucose, 1 mM MgCl_2_; pH 7.3) and platelet counts determined using a Coulter counter (Beckman Coulter). Platelets were left to rest for 30 min and count was adjusted as required using modified Tyrodes buffer.

### Platelet Spreading

For fixed cell immunofluorescence imaging, platelets were diluted to 2 × 10^7^ ml^−1^ in modified Tyrode’s buffer and allowed to spread on fibrinogen (100 µg ml^−1^) (Enzyme Research) or 95% Type I, 5% Type III (Horm) collagen (10 µg ml^−1^) (Takeda/Nycomed) coated coverslips for 45 min at 37°C, 5% CO_2_. Where indicated, platelets were pre-activated with 0.1 U ml^−1^ thrombin (Sigma) prior to plating on fibrinogen coverslips. At the end of the incubation, non-adhered platelets were removed by washing once in PBS and then fixed for 10 min in 10% neutral-buffered formalin (Sigma-Aldrich).

For resting platelet and chilled platelet imaging experiments, washed platelets at 4 × 10^7^ ml^−1^ were incubated at 37°C or 4°C for 3 h prior to fixation. A further aliquot of platelets stored at 4°C for 3 h were then re-warmed to 37°C for 30 min before fixation in an equal volume of 10% neutral-buffered formalin for 15 min. Fixed platelets were added to a 24-well plate containing poly-L-lysine (Sigma) coated glass coverslips and centrifuged to immobilize platelets.

### Immuno-labeling

Following PBS washes cells were permeabilised with 0.1% (v/v) Triton X-100 for 5 min and then washed in PBS and blocked for 30 min in block buffer (1% BSA, 2% goat serum in PBS). Tubulin was immunolabelled with 2 µg ml^−1^ anti-α-tubulin (Clone DM1A, Sigma-Aldrich) diluted in block buffer for 1 h at room temperature. Tubulin was secondary labeled with 4 µg ml^−1^ anti-mouse-Alexa647 and F-actin was labeled with 13 nM phalloidin-Alexa488 (Thermo-Fisher). Cells were washed in PBS and mounted in Hydromount (National Diagnostics) prior to imaging.

### SDS-PAGE and Western Blotting

Lysates from resting platelets were prepared from platelets at 5 × 10^8^ ml^−1^ in an equal volume of 2x lysis buffer on ice for 10 min. Lysates were mixed with sample buffer and boiled for 5 min before cooling. Samples and protein marker were loaded into wells of pre-cast polyacrylamide gels (Bolt, Invitrogen) at 60 V for 15 min to stack proteins, followed by 120 V for 1 h for separation. Proteins were transferred to PVDF membranes for 10 min using the Trans-Blot Turbo Transfer System (Bio-rad), then incubated for 1 h at room temperature in blocking buffer (5% BSA in TBST, filtered). Blots were incubated with primary antibodies against either mDia1 (Bethyl Labs, A300-078A), Fhod1 (Abcam, ab73443), or Daam1 (Clone 5D3, ECM Biosciences) at 2 μg ml^−1^ in blocking solution at 4°C overnight. Blots were washed 3 times for 10 min each in TBST, then incubated for 1 h with anti-mouse or anti-rat secondary antibody, diluted 1:10 000 in TBST, before a further 3 washes. Using LI-COR Odyssey imaging system and Image Studio software, western blots were exposed for 2–10 min.

### Platelet Aggregation and Secretion

Platelet aggregation and dense granule ATP secretion were monitored using 300 µL of washed platelets at 2 × 10^8^ mL^−1^. Stimulation of platelets with thrombin (0.03 or 0.1 U mL^−1^) or 95% Type I, 5% Type III (Horm) collagen (0.3 or 3 µg mL^−1^) was performed in a Lumi-Dual Aggro-meter (Chrono-Log Corporation) with continuous stirring at 1200 rpm at 37°C. ATP secretion was determined during aggregation using Chrono-Lume reagent (Chrono-Log Corporation).

### In Vitro Flow Studies

For flow adhesion studies, mouse blood was drawn into sodium heparin (10 IU ml^−1^) and PPACK (40 μM). Vena8 Fluoro+ biochips (Cellix) were coated with 100 μg ml^−1^ 95% Type I, 5% Type III (Horm) collagen, (Nycomed, Munich, Germany) for 1 h at room temperature. The chips were washed and blocked with PBS containing 5 mg ml^−1^ BSA for 1 h at room temperature. Anticoagulated whole blood, labeled with DiOC6 (final concentration 2 µM) for 10 min was perfused through the chamber for 6 min at a wall shear rate of 1000 s^−1^, followed by washing for 3 min at the same shear rate with modified Tyrodes buffer. Ten FOV were captured along the length of the biochip and the percentage surface area coverage calculated using Fiji [21]. Images were background subtracted, and then converted into binary images using the Otsu threshold function. Measurement of the platelet aggregates in each image was obtained using the Analyze Particles function and overall surface area coverage in each image calculated by summing the individual thrombi.

### Flow Cytometry

The receptor levels on the surface of resting platelets were determined by labeling 10 μL of washed platelets at 2 × 10^8^ ml^−1^ with 50 μL of Tyrodes containing 5 μl of the relevant FITC conjugated antibody (i.e. CD41, GPVI, CD49b, Clec2 or GPIbα; Emfret Analytics) and incubating in the dark for 30 min. Samples were diluted with 200 μl of modified Tyrodes buffer and analyzed using an Accuri C6 flow cytometer (BD Biosciences). Initial gating of platelets was performed using Forward Scatter/Side Scatter plots and then the fluorescence intensity (in FL1 channel) of 10,000 platelets in this gate was recorded for each sample. An isotype control antibody was used to subtract background and values presented as mean fluorescence intensity (MFI).

For *P*-selectin exposure and fibrinogen binding assays, platelets were stimulated with 0.1 U ml^−1^ thrombin for 2 min at 37°C or with PBS (unstimulated controls) before staining with either anti-*P*-selectin-FITC or Alexa488-fibrinogen for 30 min and flow cytometry as described above.

### Clot Retraction

Whole murine blood was anti-coagulated with sodium citrate and PRP prepared as above. The platelet count was adjusted to 3 × 10^8^ ml^−1^ with modified Tyrodes Buffer containing CaCl_2_ (2 mM) and fibrinogen (2 mg ml^−1^). 400 μl of this mix was placed into an aggregometer tube and incubated at 37°C for 5 min. 2 μl of mouse erythrocytes were added for color contrast. Thrombin (10 U ml^−1^) was added and mixed with a paperclip and clot retraction was allowed to proceed at 37°C for 1 hour with the paperclip present. At appropriate time points, the clot was pulled out with the paperclip and the remaining serum volume measured.

### Platelet Turnover and Immune-induced Thrombocytopenia

Platelet turnover was measured by injecting mice with 0.1 μg g^−1^ body weight of GPIbβ DyLight488 conjugated antibody (Emfret Analytics; X488). Blood samples were taken from the tail vein at various time points after injection (1, 24, 48, 72, 96, and 120 h) and the percentage of DyLight488 labeled platelets was measured using an Accuri C6 flow cytometer.

To establish the dynamics of platelet production in these mice, platelets were depleted and their recovery monitored. A baseline platelet count was established by tail vein bleeds, after which immune-induced thrombocytopenia was induced in mice by tail vein injection of 2 μg g^−1^ body weight anti-GPIbα antibody (Emfret analytics; R300) followed by blood sampling at 1, 3, 5, 7 10 and 14 days post-injection to determine the platelet recovery using an ABX Micros Pentra 60 hematological analyzer.

### Bone Marrow Megakaryocyte Isolation, DNA Ploidy Measurement and Megakaryocyte Spreading Assays

Bone marrow megakaryocyte progenitor cells were isolated and cultured in vitro and mature megakaryocytes were enriched for via a BSA gradient as previously described [22]. For DNA ploidy measurements mature megakaryocytes were stained with anti-CD41-FITC (MWReg30, BD Biosciences), fixed in 0.5% PFA, and DNA labeled with propidium iodide overnight (PBS, 2 mM MgCl_2_, 0.05% saponin, 0.01 mg ml^−1^ propidium iodide and 10 U ml^−1^ RNAse). For DNA ploidy measurements of freshly isolated megakaryocytes, whole bone marrow was flushed from the femur and tibia in flushing buffer (PBS, 10% EDTA, 10% BSA, 1 M HEPES) and dissociated cells were stained with anti-CD42-FITC (Xia-H10, Emfret Analytics) in staining buffer (PBS, 2,5% FBS, 5% EDTA, 5% BSA, 0.5 M HEPES), before DNA labeling as above. Cells were analyzed using a FACSCalibur flow cytometer (Becton Dickinson) by initially gating on the large granular population on FSC/SSC plots and then measuring the propidium iodide intensity of CD41/CD42 positive cells (Supp. 5C). Analysis was performed using FlowJo software.

For megakaryocyte spreading analysis, mature megakaryocytes were allowed to spread on fibrinogen coated coverslips in the presence of SDF1α (0.25 μg mL^−1^; Peprotech) at 37°C/5% CO_2_ for 1 or 3 h, before fixing and immuno-labeling as described above. For proplatelet, formation assays heparin (70 U mL^−1^) was added during spreading.

### Histology of Mouse Femur

Dissected mouse femurs were incubated overnight in 4% neutral-buffered saline containing 5 mM sucrose. Fixed femurs were decalcified in 10% EDTA/PBS and then processed for paraffin embedding. 10 µm longitudinal sections were stained with hematoxylin and eosin and imaged using a Zeiss Axio Scan.Z1. Megakaryocytes within the bone marrow were identified by morphology and size. For each section, all megakaryocytes within the diaphysis of the femur were counted manually and results are presented as number of megakaryocytes per mm^2^.

### Microscopy

Images were acquired using an Axio Observer 7 inverted epifluorescence microscope (Carl Zeiss) with Definite Focus 2 autofocus, Colibri 7 LED illumination source, Hammamatsu Flash 4 V2 sCMOS camera, Filter sets 38 and 50 for Alexa488 and Alexa647, respectively, and DIC optics. Acquisition was performed using Zen 2.3 Pro software. For platelet imaging, a 63 × 1.4 NA oil immersion objective lens was used and for megakaryocyte imaging a 20 × 0.8NA air objective lens was used. LED power and exposure times were chosen to be appropriate for each set of samples but kept the same within each experiment. For platelet spreading experiments, five image stacks were taken per genotype from three independent experiments. For resting platelet experiments, five image stacks were taken per genotype from five independent experiments. For megakaryocyte spreading, 38–50 cells were captured per coverslip. For proplatelet formation assays, a tiled image (6000 x 6000 μm) was captured per sample. For SR-SIM imaging, samples were imaged on a Nikon N-SIM-S microscope with Ti2-E inverted microsope stand, PFS4 focus stability system, 100 × 1.49 NA oil immersion objective lens with automatic correction collar adjustment, LU-NV-L laser bed, Hammamatsu Orca Flash 4 V3 sCMOS camera, TI2-FT N-SIM Motorized Filter set, and NIS-AR elements V5 software. Additional image processing and measurements were performed using Fiji.

### Image Analysis

Post-capture image analysis was performed using Fiji. For fixed cell measurements (surface area of spread or resting cells) data are from means from three or five independent experiments, respectively, with between 150 and 350 individual platelets analyzed for each treatment per experiment. The spread platelet and megakaryocyte area were measured using thresholding, ROI manager and Measure functions of Fiji. Distribution of platelet-spreading morphology and the classification of proplatelet forming megakaryocytes was performed using a semi-automated workflow [23].

### Statistics

Data analysis was carried out using Microsoft Excel and Graphpad Prism V6. Results are shown as mean ± SD, number of replicates (unless indicated), and statistical significance was analyzed using ANOVA or Kruskal–Wallis one-way analysis of variance (after determining whether data were normally distributed or not) and Dunnetts multiple comparison tests–effects were compared to WT. *P* values are given in the text whilst in figures is represented by stars compared to WT (*P* < .05 = *; *P* < .01 = **; *P* < .005 = ***; *P* < .001 = ****).

## Results

### Mouse Models

We have previously reported that platelets from an mDia1 knockout mouse model have no platelet function phenotype [16]. However, reports from shRNA knockdown of mDia1 in human CD34^+^ cells and patients expressing a gain of function mutation in mDia1 demonstrate an effect on platelet formation [14,24]. Furthermore, we have recently demonstrated the importance of formin FH2 domains for platelet function through use of a global FH2 domain inhibitor [17]. Therefore, we generated a transgenic mouse model for the most abundant platelet formin, Fhod1 (Fhod1 KO), which we have previously shown is activated upon platelet stimulation [16], and compared platelet function and formation to wild type (WT) mice. We also compared them to platelets and megakaryocytes from the mDia1 transgenic model (mDia1 KO) and a double mDia1/Fhod1 transgenic model (DKO) to investigate redundancy between the two formin proteins. Outwardly, all mouse models (mdia1KO, Fhod1 KO, and DKO) displayed no overt phenotype and inheritance of the mDia1 and Fhod1 alleles was Mendelian (Supp. 2B).

### Hematological Analysis of Knockout Mice

**Figure 1.**
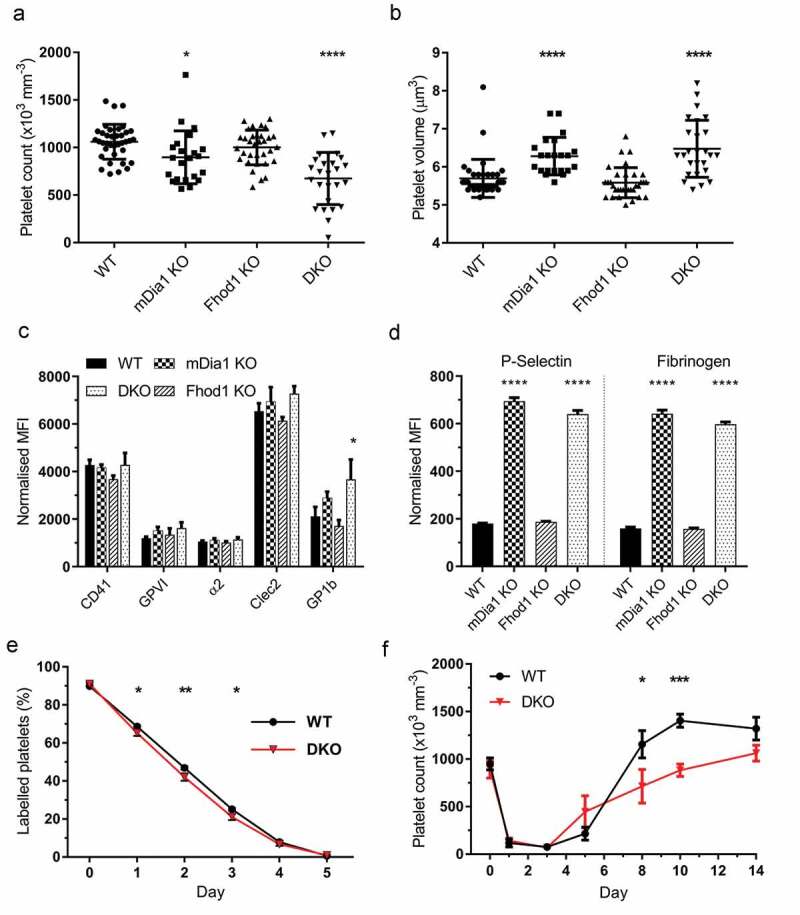
**Loss of mDia1 and Fhod1 affects platelet production and turnover**. a) Whole blood platelet counts and b) mean platelet volume was determined for all genotypes. mDia1 KO and DKO mice displayed a macrothrombocytopenia which was more severe in the DKO mice. Each data point represents 1 mouse. c) DKO platelets had a significant increase in surface GPIbα levels. No significant difference was observed for other platelet surface receptors (n = 4). d) Resting platelets from both the mDia1 KO and DKO mice had significantly elevated levels of *P*-selectin exposure on their surface and enhanced fibrinogen binding compared to WT and Fhod1 KOs (n = 4). e) Platelet lifespan (n = 4) and f) platelet recovery from immune-induced thrombocytopenia (n = 6) indicated that DKO platelets have a significantly increased rate of clearance and a significantly decreased recovery compared to WT mice. Error bars represent mean ± SD for a) and b) and ± SEM for c-f)

Detailed analysis of the hematological parameters of Fhod1 KO mice demonstrated that blood cell production in these mice was normal. They displayed a normal platelet count and platelet volume when compared to WT mice (Figure 1a & 1b), and the total number of white blood cells, and the proportion of each white blood cell type was normal (Supp. 3A – F).

In contrast, both mDia1 KO and DKO mice displayed altered platelet count and platelet volume (Figure 1& b). mDia1 KO mice had large platelets (*P* < .0001) with a small, but significant decrease in platelet number (*P* = .011) whilst DKO mice developed a more severe macrothrombocytopenia (37% reduction) with significantly decreased platelet number (*P* < .0001) and increased platelet volume (*P* < .0001). The loss of mDia1 has previously been reported to affect white blood cell numbers [19,25–27]; however, no differences were seen in our mDia1 mice. We did, however, observe a significant reduction in total white blood cell count in DKO mice compared to WT (*P* = .034) (Supp. . 3A). Further investigation indicated that in DKO mice the percentage of lymphocytes was significantly reduced (*P* = .0025), and the percentage of neutrophils was significantly increased (*P* = .0063) compared to WT. Levels of monocytes, basophiles and eosinophils were unaltered. This data indicates a role for the formins mDia1 and Fhod1 in maintenance of platelet number and volume, and in the proper development of WBCs.

### Analysis of Resting Platelets

As we had observed a disruption in platelet number and volume, we investigated the resting platelet phenotype further. Analysis of the surface receptor expression profile by flow cytometry showed that, consistent with the hematological analysis, Fhod1 KO platelets were similar to WT in terms of CD41, CPVI, CD49b, CLEC2, and GPIbα expression (Figure 1c). Likewise, mDia1 KO platelets were indistinguishable from WT in terms of their surface receptor profile, despite having an increased platelet volume. In contrast, resting platelets from the DKO mice had significantly elevated levels of GPIbα on their surface compared to WT (*P* = .028) and Fhod1 KO (*P* = .002), although other receptor levels were similar to WT (Figure 1c, Supp. . 4A). Surface GPIbα levels on mDia1 KO platelets were intermediate between WT and DKO but not significantly different to either. Further investigation of the resting platelets showed that both mDia1 KO and DKO platelets had elevated surface levels of *P*-selectin (*P* < .0001) and increased fibrinogen binding capacity (*P* < .0001) compared to WT mice (Figure 1d).

### Analysis of Platelet Clearance

As GPIbα has been implicated in mediating platelet clearance from the circulation [28], and it is believed that *P*-selectin surface expression may enhance platelet clearance by leukocytes [29], we monitored the platelet population half-life in these mice. Whilst mDia1 KO and Fhod1 KO mice almost exactly overlaid the WT (Supp. B), we observed a very small, but significant increase in platelet clearance for DKO mice at day 2 (*P* = .0026) and day 3 (*P* = .0146) when compared to WT (Figure 1e). This suggests that the increased GPIbα levels and not the elevated *P*-selectin on resting platelets that, at least in part, is responsible for the observed thrombocytopenia in the DKO mice.

### Analysis of Platelet Production

Thrombocytopenia can also be caused by a reduction in platelet production. As mutations in mDia1 have previously been implicated in reduced proplatelet formation [14,24] we monitored platelet recovery following immune-induced thrombocytopenia. All genotypes displayed a rapid reduction in platelet count and subsequent recovery with no significant difference observed between either mDia1 KO or Fhod1 KO mice and WTs (Supp. 4C). However, DKO mice did not display a rebound increase as seen in WT mice resulting in a significant reduction in platelet count at both day 8 (*P* = .031) and day 10 (*P* = .0003) post depletion (Figure 1f). Although this is a mild reduction in platelet production, a combination of a slight increase in clearance rate is likely to account for the reduction in platelet number observed in the DKO mice.

### Effect of Loss of Formins on Megakaryocytes

**Figure 2.**
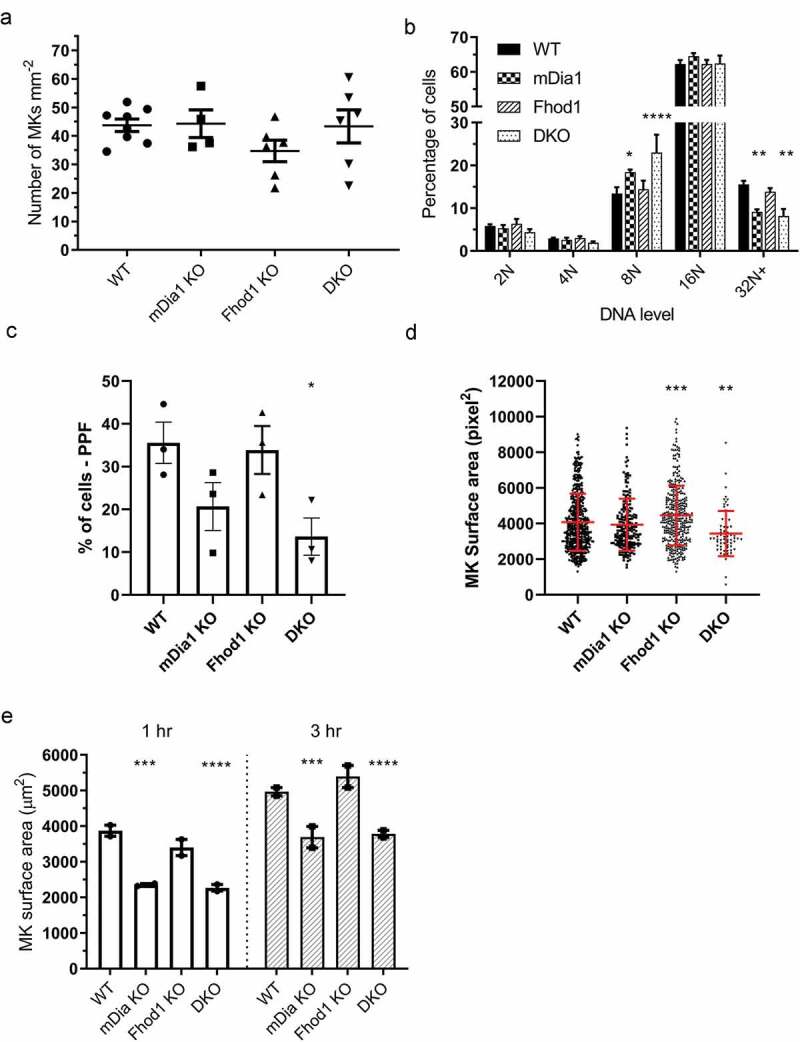
**Loss of mDia1 and Fhod1 causes a developmental defect in bone marrow megakaryocytes**. a) Counts of megakaryocytes in bone marrow of femur sections showed no significant difference between any of the genotypes tested. Each data point represents 1 mouse. b) The DNA ploidy level of megakaryocytes freshly isolated from bone marrow showed that both mDia1 KO and DKO mice had significantly increased levels of 8 N cells and significantly decreased levels of 32 N+ cells in their bone marrow (n = 3). c) The percentage of cells classified as proplatelet forming (PPF) by image analysis was reduced in both mDia1 and DKO mice (n = 3). d) The surface area of PPF cell is reduced in DKO mice. Each data point represents 1 cell (pooled data from the 3 mice). e) Surface area quantification of mature megakaryocytes spread for 1 and 3 h indicated that both mDia1 KO and DKO cells has a reduced ability to spread (n = 2). Error bars represent mean ± SEM for a), b), c) & e) and mean ± SD for d)

To further investigate the effects of loss of these proteins on platelet production and megakaryocyte development, we prepared bone marrow sections from the femurs of these mice and counted the number of mature megakaryocytes in these sections. We observed no significant difference between the four genotypes in the number of megakaryocytes observed (Figure 2a, Supp. . 5A).

In light of this, we further characterized bone marrow megakaryocyte development by isolating mature megakaryocytes from bone marrow of the femur and tibia of the mice. Firstly, the proportion of bone marrow cells that were identified as megakaryocytes reflected the counts made from bone femur sections and no significant difference between the genotypes was observed (*P* = .065) (Supp. . 5B). To characterize megakaryocyte development, we performed DNA ploidy measurements on these freshly isolated megakaryocytes. For Fhod1 KO mice, as expected from the normal platelet counts, no difference was observed in MK ploidy compared to WT (Figure 2b, Supp. 5D-E). However, for both mDia1 KO and DKO mice, there was a significant increase in the proportion of 8 N megakaryocyte (mDia1 *P* = .04, DKO *P* = .0001) and a significant decrease in the proportion of 32 N+ megakaryocytes (mDia1 *P* = .0071, DKO *P* = .0019) compared to WT (Figure 2b). This indicates that whilst there is no difference in the total numbers of megakaryocytes in the bone marrow, megakaryocytes differentiation, as measured by DNA ploidy, is reduced.

To investigate this effect further we isolated lineage negative bone marrow cells and differentiated them *in vitro* before measuring DNA ploidy profiles. However, megakaryocyte differentiation and DNA ploidy in *in vitro* cultured megakaryocytes showed no major differences between the genotypes (Supp. 5F), indicating that formins may be important in sensing aspects of the bone marrow environment *in vivo*.

The ability of megakaryocytes to undergo proplatelet formation was assessed using proplatelet assays and an image analysis workflow [23] (Supp. . 6A-C). For Fhod1 KO cells, no difference in the % of cells classified as proplatelet forming compared to WT was observed (Figure 2c). Interestingly, the surface area of proplatelet forming cells was significantly increased compared to WT (*P* = .0003, Figure 2d). In line with the observed thrombocytopenia, both mdia1 KO and DKO megakaryocytes showed a reduction in the number of cells undergoing proplatelet formation (Figure 2c) although this was only significant for the DKO (*P* = .04). DKO cells also displayed a significant reduction in the surface area of proplatelet forming cells (*P* = .0054, Figure 2d). This reduction in proplatelet formation, along with the increase in platelet clearance, may underlie the observed thrombocytopenia in these mice.

To test if loss of formin proteins has an effect on the ability of megakaryocytes to organize their actin and hence their ability to spread, mature megakaryocytes were allowed to spread on fibrinogen for 1 or 3 h. The average surface area of both mDia1 KO and DKO cells was reduced compared to controls at both 1 h (mDia1 KO *P* = .0012, DKO *P* = .0008) and 3 h (mDia1 KO *P* = .0016, DKO *P* = .0003) (Figure 2e & Supp. 6D). This, therefore, indicates that megakaryocyte spreading on fibrinogen is partially formin mediated. Although spreading is not required for platelet production *in vivo*, this suggests that mDia1 and Fhod1 are required for normal megakaryocyte actin organization during platelet production.

Taking the data all together indicates that mDia1 KO and DKO mice have decreased platelet count, increased platelet size, and increased surface levels of GPIbα and platelet activation markers. DKO mice have altered kinetics of platelet count, namely, slightly increased platelet clearance and delayed platelet recovery. Therefore, it is likely that formins play a role in mediating megakaryocyte cytoskeletal dynamics and differentiation *in vivo* which contributes to the observed macrothrombocytopenia.

### Role of Formins in Regulating the Platelet Cytoskeleton

**Figure 3.**
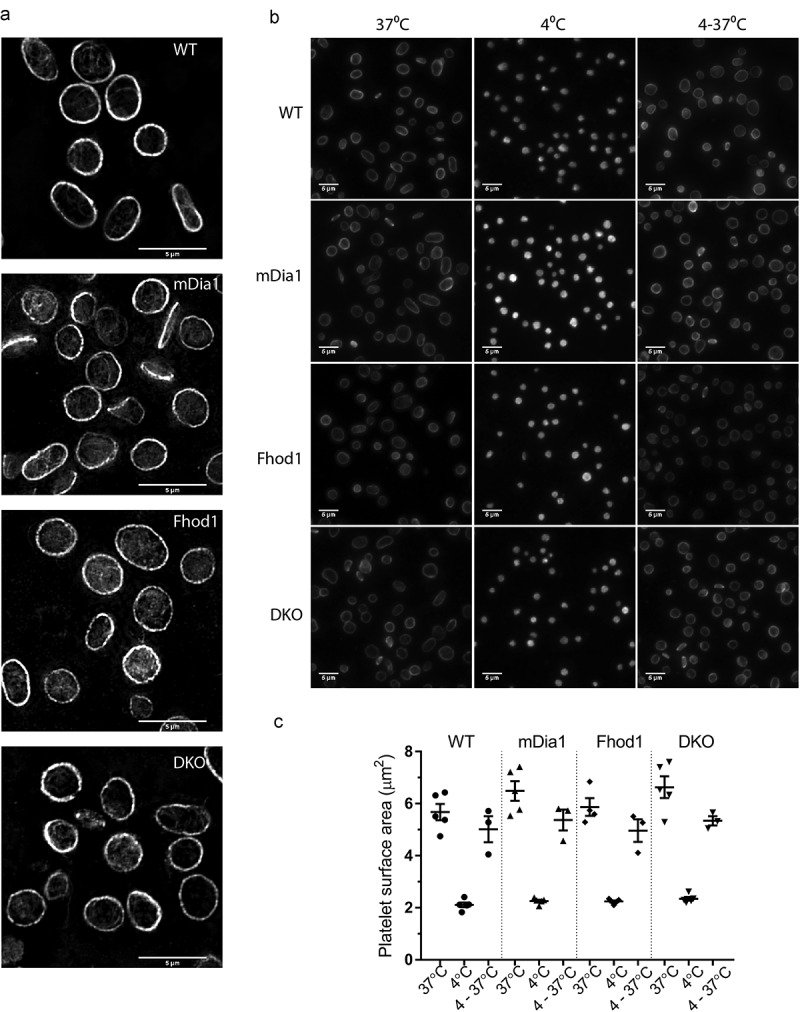
**Loss of mDia1 and Fhod1 has no effect on cold-induced microtubule ring depolymerization**. a) Representative SIM images of the microtubule coils of resting platelets. b) Resting platelets from WT, mDia1 KO, Fhod1 KO or DKO mice were stored at either 37°C or 4°C for 3 h. A subsample was also re-warmed to 37°C for 30 min. After fixing platelets were labeled for tubulin and b) resting platelet surface area was determined. Each data point represents 1 mouse. Scale bars = 5 μm. Error bars represent mean ± SEM

We have recently showed that blocking formin-mediated actin polymerization using a selective inhibitor blocks platelet spreading and causes increased coiling of the platelet microtubule coil [17]. To establish the effect that loss of specific formin proteins have on the organization of the resting platelet cytoskeleton, we stained fixed, resting platelets with α-tubulin antibodies and used SIM imaging to visualize the microtubule coil. WT platelets displayed a distinct coil at the periphery of the platelet, and evidence for microtubules extending throughout the cytoplasm of the platelet (Figure 3a, top panel). Resting platelets from mDia1 KO, Fhod1 KO, and DKO mice also displayed microtubule coils, although qualitatively, the staining gave a more “patchy” pattern than in WT platelets and there was evidence for more disorganized microtubules within the center of the platelets, especially in Fhod1 KO platelets (Figure 3a, Lower three panels). However, this disruption was not as evident as that observed in platelets of human patients with a DIAPH1 gain of function mutation [14]. An additional feature describing platelets of patients with DIAPH gain of function was that the microtubule coil was protected from depolymerization upon chilling. To test this we stored platelets from our mouse models at either 37°C or 4°C prior to fixation and then stained them for microtubules and imaged using widefield fluorescence microscopy (Figure 3b). The surface area of resting platelets, outlined by the microtubule coil, confirmed the increased size of mDia1 KO and DKO platelets compared to WT and Fhod1 KO cells. (Figure 3c). As expected, the surface area of platelets chilled at 4°C for 3 h was much smaller than platelets stored at 37°C for all genotypes. There was no significant difference between the genotypes at 4°C (Figure 3c). Qualitative analysis of the images from these experiments showed that although occasional platelets with residual coils could be identified, the microtubule coils of mDia1 KO, Fhod1 KO and DKO mice generally depolymerized to the same extent as WT (Figure 3b). Rewarming of chilled platelets to 37°C allowed re-polymerization of the microtubule coil in all genotypes (Figure 3b). Interestingly, the rewarmed platelets did not return to their original size and surface areas were slightly reduced compared to un-chilled resting platelets (Figure 3c). In addition, the differences observed between mDia1 KO and DKO and WT platelets were lost. Together this indicates that the microtubules in these cells are “normal” and any difference in resting platelet size is likely due to issues in platelet production or in how motor proteins interact with the coil.

**Figure 4.**
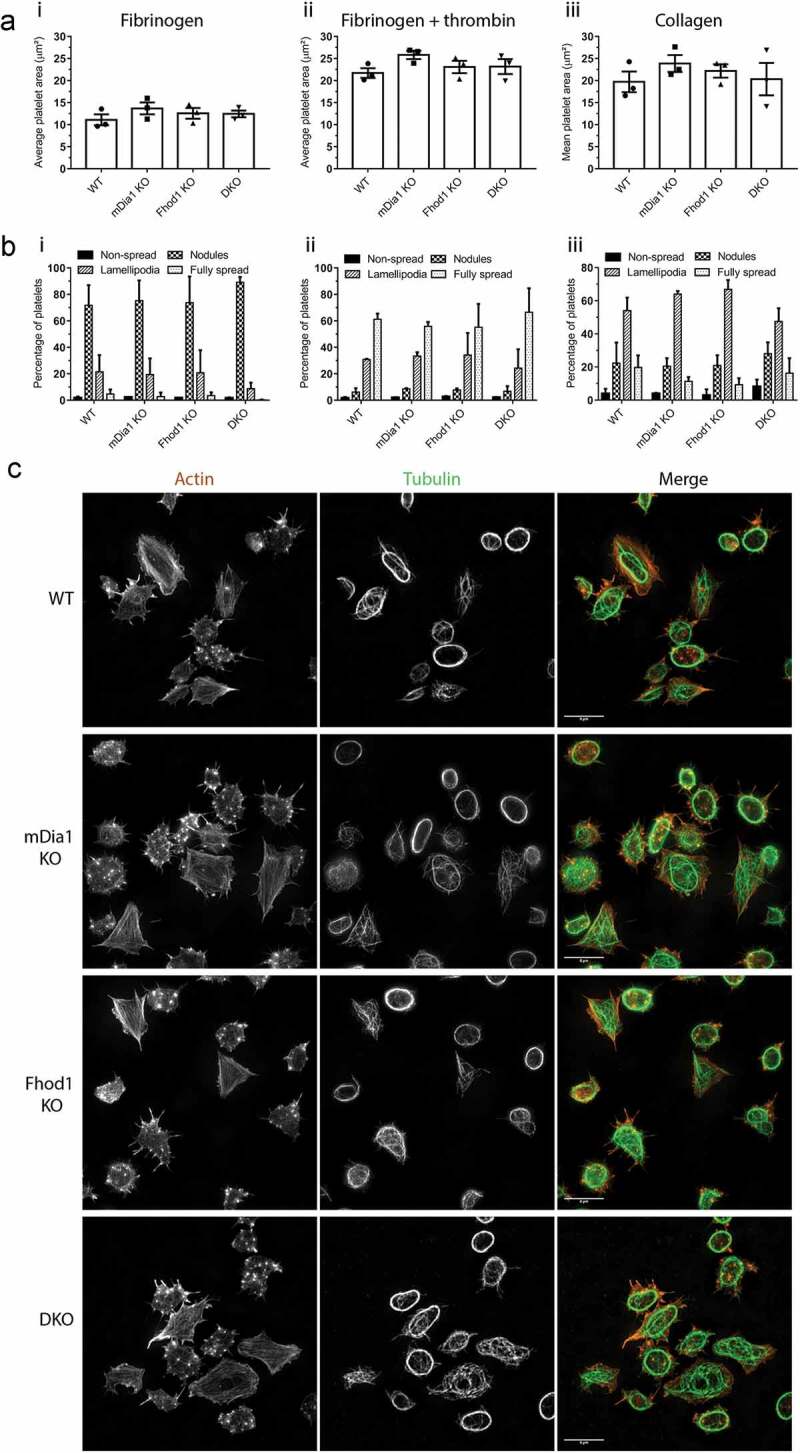
**Loss of mDia1 and Fhod1 has no effect on platelet spreading**. a) Analysis of spread platelet surface area and b) spread platelet morphology of washed platelets from WT, mDia1 KO, Fhod1 KO or DKO mice spread for 45 min on (i) fibrinogen, (ii) fibrinogen + thrombin and (iii) collagen (n = 4). No significant difference in surface area or morphology was observed. c) Representative images of platelets spread on fibrinogen for 45 min, fixed, stained for actin and tubulin, and imaged using SR-SIM. Scale bars = 5 μm. Error bars represent mean ± SEM in A) mean ± SD in B)

Formins are actin nucleation-promoting factors and we have previously demonstrated the role of FH2 domains in regulating platelet spreading [17]. Therefore, we monitored the effect of the loss of mDia1, Fhod1, or both proteins on platelets spreading. Confirming our previous studies [16], the loss of mDia1 has no effect on platelet spreading on any of the surfaces tested. Similarly and surprisingly, knockout of Fhod1 or both mDia1 and Fhod1, had no effect on platelet spreading in terms of spread surface area (Figure 4a) or in the distribution of spreading morphologies displayed (Figure 4b). Furthermore, SR-SIM imaging of F-actin and microtubules demonstrates that platelets from all genotypes are capable of forming actin nodules, filopodia, lamellipodia, stress fibers, and that microtubule coils reorganize to form characteristics spread platelet networks (Figure 4c, Supp. . 7).

### Role of Formins in Regulating Platelet Function

Platelets from the knockout models were compared to WT platelets in a range of platelet function assays. No significant differences were observed between WT and mDia1 KO, Fhod1 KO, or DKO platelets in aggregation, ATP secretion, *P*-selectin exposure, fibrinogen binding, aggregation to collagen under flow, or clot retraction. A summary of the data for these experiments can be found in Table I and Supp. . 8.

**Table I.** Summary of platelet function testing data

(Mean ± SEM)	WT	mDia1 KO	Fhod1	DKO
**Aggregation** **(% at 5 min)** Coll 0.3 μg ml^−1^ Coll 3 μg ml^−1^ Thr 0.03 U ml^−1^ Thr 0.1 U ml^−1^	18.7 ± 7.1 61.9 ± 4.1 7.2 ± 4.8 65.0 ± 3.3	21.8 ± 9.9 54.6 ± 11.9 7.2 ± 6.9 65.0 ± 3.4	10.0 ± 4.1 46.8 ± 8.3 6.9 ± 5.8 59.2 ± 4.2	20.5 ± 5.1 62.6 ± 4.7 13.9 ± 8.0 54.1 ± 6.1
**ATP Secretion** **(nmol)** Coll 0.3 μg ml^−1^ Coll 3 μg ml^−1^ Thr 0.03 U ml^−1^ Thr 0.1 U ml^−1^	0.114 ± 0.04 0.979 ± 0.20 0.004 ± 0.004 1.181 ± 0.28	0.138 ± 0.06 0.638 ± 0.17 ND 0.950 ± 0.44	0.020 ± 0.02 1.150 ± 0.29 ND 1.319 ± 0.34	0.092 ± 0.04 0.878 ± 0.09 0.005 ± 0.004 1.360 ± 0.89
**P-selectin expression** **(MFI)** Thrombin stimulated	1975.7 ± 689.5	2825.5 ± 345.7	1811.0 ± 202.4	2319.1 ± 402.4
**Fibrinogen binding** **(MFI)** Thrombin stimulated	1319 ± 515	3139 ± 1309	2135 ± 1075	1875 ± 612
**Aggregation under flow (surface area, μm^2^)** Collagen @ 1000s^−1^	8759 ± 1981	8732 ± 1935	10 895 ± 1595	11 999 ± 2617
**Clot retraction** **(weight of liquid, g)** 10 min 20 min 30 min	0.175 ± 0.006 0.237 ± 0.010 0.276 ± 0.002	NA NA NA	NA NA NA	0.191 ± 0.003 0.247 ± 0.014 0.274 ± 0.009

ND – not detected; NA – not performed

Taken together this data indicates that mouse platelets can tolerate the loss of both mDia1 and Fhod1, indicating that formin function in platelets can be sufficiently covered by another formin family member, or more generally by other actin regulatory pathways during platelet activation.

## Discussion

In this manuscript, we report the platelet and MK phenotype of a Fhod1 KO and a double Fhod1 and mdia1 KO mouse model. These mice are compared to the previously characterized mDia1 KO mice [16] and to WT mice.

The clearest defect observed is disrupted platelet production with a decreased platelet count being observed, which is most severe in the DKO mice. The evidence presented here suggests that mDia1 is the major player in this phenotype, as mDia1 KOs show a tendency toward thrombocytopenia, which is exacerbated by the loss of Fhod1 in the DKOs. In contrast, Fhod1 KO mice alone display no change in platelet number strongly suggesting that Fhod1 contributes, but does not play a major role in this process. In addition to the reduced production of platelets in mDia1 KO and DKO mice, there are also disruptions to the process which results in enlarged platelet size, and perturbed surface receptor profiles.

The reason for thrombocytopenia in these mice is likely to be multi-factorial and a combination of both decreased production and increased clearance. We demonstrated that DKO mice have disrupted megakaryocyte development and platelet production, data which is in line with that from gain of function mutations and knockout of DIAPH1 [14,24]. The mechanisms underlying normal proplatelet formation are complex, but it is clear that both the microtubule and actin cytoskeletons are required [6]. Recent work has identified that both post-translational modifications of tubulin [30,31] and interactions between cytoskeletal motors, the cytoskeleton, and response to the environment (e.g. flow) [32] modulate platelet formation. Formins have important roles to play in polymerization of actin filaments, organizing actin and microtubules [13], and in regulating both actin and tubulin post-translation modification [33,34] and so the effects on proplatelet formation is likely to be based on multiple mechanisms.

Alongside the changes in platelet formation, the observed increased basal expression of GPIbα could enhance CR3-mediated macrophage clearance of platelets in the liver [28], which along with increased *P*-selectin-mediated clearance of resting platelets by neutrophils [29,35], could contribute to the observed thrombocytopenia. Platelet half-life experiments would seem to support this hypothesis as DKO mice have a very small, but significant increase in platelet clearance. Interestingly, mDia1 KO mice showed similar kinetics of platelet clearance to WT mice (data not shown). mDia1 KO platelets showed a similar level of *P*-selectin expression as DKO platelets, but a reduced GPIbα expression, indicating that it is the enhanced GPIbα levels that are the primary driver of the observed platelet clearance in DKO mice. Our observation that neutrophil proportion is increased in DKO, but not mDia1 KO mice seems at odds with this; however, it has been reported that aberrant formin activity can give rise to myeloproliferative defects [27], and therefore the situation with regards to altered leukocyte populations may be complex.

Increased GPIbα expression on platelets might also be expected to enhance initial binding to Von Willebrand factor upon exposure to collagen. However, *in vitro* flow experiments of whole blood over collagen-coated chambers indicated no significant effect of this increased GPIbα on aggregate formation (Table I and Supp. . 8). Whether this small increase in GPIbα is sufficient to cause reduced time to clot *in vivo*, is unclear. Furthermore, increased expression of *P*-selectin on resting platelets from mDia1 KO and DKO mice might indicate enhanced alpha granule secretion; indeed *P*-selectin expression in activated platelets was increased in these mice; however, this was not significantly different to WT. Enhanced alpha granule secretion would lead to enhanced release of pro-thrombotic and pro-inflammatory mediators leading to the formation of platelet micro-aggregates or platelet-leukocyte, monocyte, and neutrophil aggregates and increased thrombotic risk. However, we did not test the levels of these factors directly in our mice.

We also investigated whether MK development and platelet production were affected by the loss of mDia1 and Fhod1. Previous studies have indicated that disruption to mDia1 (either knockdown, or gain of function) can disrupt this process [14,24]. Whilst we observed normal numbers of MKs in bone marrow sections, we report a reduction in MK spreading and the level of polyploidization in mDia1 KO and DKO mice. These data indicated that both mDia1 and Fhod1 activity in MKs is required for normal development and actin dynamics. Interestingly, this difference in ploidy was only observed in MKs freshly isolated from bone marrow and not when lineage negative cells were differentiated *in vitro*. RhoA/mDia1 has been shown to play a role in crosstalk between force sensing and signal transduction pathways [36]. Whilst this might indicate a possible role for formins in the mechanosensing or mechanotransduction of environmental signals to the developing MK that modulate differentiation, the reason for the lack of consistency between freshly isolated and *ex vivo* differentiated MKs remains unclear.

It is obvious that the role of Fhod1 in MKs and platelets is limited, compared to that for mDia1. This is supported by our observations that Fhod1 KO mice are more like WT mice than mDia1 KO or DKO. Fhod1 has been demonstrated to be required for stress fiber and podosome formation and dynamics in several cell types [37–39]. It has also been shown to be required for proliferation, epithelial-mesenchymal transition, tumor growth, invasion, and cell migration in several cancer lines [40–42]. We would have therefore expected Fhod1 might be of relevance in MKs, especially in the context of MK and proplatelet protrusion into the vasculature. It would appear from our data; however, that this is not the case, or this function can adequately be maintained by other actin regulatory proteins.

A particularly surprising outcome from the DKO mice is the lack of any clear functional platelet defect. Considering the potent roles for formin proteins in other cell types, the most likely explanation for the lack of effect here is redundancy. Our previous data mining of the mouse platelet proteome [13,43] indicated that there are 4 formin proteins expressed in mouse platelets; Daam1 (8529 copies, 14.5% of total formin expression), mDia1 (14 408, 24.5%), Fhod1 (21 598, 36.7%) and Inf2 (14 269, 24.3%). Therefore, DKO platelets still retain approximately 40% of their formin protein content, albeit with a different protein profile. We saw no evidence for the up-regulation of Daam1 in our mice (Supp. 2B). Although different formin proteins have been associated with different cellular functions [44], the key characteristic of a formin is the presence of FH1 & FH2 domains which together mediate the nucleation and elongation of actin filaments [45]. It is probable that 40% formin mediated-actin polymerizing activity, alongside redundancy between actin polymerization regulatory pathways, is sufficient for “normal” platelet function. Indeed, there are examples of formin redundancy recorded in the literature [46], and this hypothesis fits with our observation of a dose-dependent effect on platelet spreading of the FH2 domain inhibitor SMIFH2 [17]. It is interesting to note that in patients with a DIAPH1 gain of function mutation, a similar effect was observed; namely, disrupted platelet production, but a relatively mild platelet functional defect [14], and in Arp2/3 knockout mice, twinfilin/cofilin1 double knockout mice and ADF/cofilin knockout mouse, similar discrepancies between alterations to platelet production and mildly affected hemostasis are observed [47–49].

Further evidence to support this notion can be found by looking in more detail at the molecular function of the two remaining proteins, Daam1 and Inf2. Daam1 is released from its auto-inhibited state by the combined actions of Disheveled (Dvl) and RhoA [50]. RhoA is activated upon platelet stimulation (reviewed by [51]), and work by Steele *et al*. demonstrated that upon platelet activation Dvl associated with Daam1. Therefore, it is highly likely that Damm1 would be contributing to actin polymerization in activated platelets. To the best of our knowledge, Inf2 has not been studied in platelets and so the contribution of this formin to platelet organization is unclear. However, biochemical studies on Inf2 indicate that it contains a WH2 domain which is involved in mediating its auto-inhibition [52] and that it is binding of actin monomers to this domain that relieves the auto-inhibition [53]. The initial increase in actin filament severing and depolymerization immediately upon platelet activation could therefore provide an environment for the activation of Inf2 and subsequent nucleation/elongation of new actin filaments. A further intriguing possibility is the observation that Inf2 can regulate the activity of mDia1 [54,55] and that this might be important for modulating the interaction of mDia1 with microtubules [56]. The implications of this for platelets remain unclear, especially in the absence of mDia1 and Fhod1, but it would be interesting to study the role of Inf2 and Daam1 in platelet function further.

In conclusion, we have shown that loss of mDia1 and Fhod1 causes a reduction in platelet formation and may disrupt MK development. In contrast platelet function is more tolerant of loss of these two formin family members, as in the milieu of an activated platelet, the formin-mediated actin polymerization requirements can be met by other expressed formins. It would therefore be interesting in future studies to investigate the contributions of Daam1 and Inf2 to MK and platelet function.

## Supplementary Material

Supplemental Material
