# Supplementary material for: Loss of mDia1 and Fhod1 impacts platelet formation but not platelet function
Source: Platelets. 2020 Sep 27;32(8):1051–62. doi: 10.1080/09537104.2020.1822522 (PMC8635707; doi:10.1080/09537104.2020.1822522)
Supplement: Supplemental Material [file IPLT_A_1822522_SM3010.docx]

Loss of mDia1 and Fhod1 impacts platelet formation but not platelet function.

Malou Zuidscherwoude^ab^, Elizabeth J. Haining^a^, Victoria A. Simms^a^, Stephanie Watson^a^, Beata Grygielska^a^, Alex T. Hardy^a^, Andrea Bacon^c^, Stephen P. Watson^ab^ and Steven G. Thomas^ab^*

^a^Institute of Cardiovascular Sciences, College of Medical and Dental Sciences, University of Birmingham, Edgbaston, Birmingham, B15 2TT, UK

^b^Centre of Membrane Proteins and Receptors (COMPARE), University of Birmingham and University of Nottingham, Midlands, UK

^c^Genome Editing Facility, Technology Hub, College of Medical and Dental Sciences, University of Birmingham, Edgbaston, Birmingham, UK.

* Corresponding author

Steven G. Thomas,

Institute of Cardiovascular Sciences,

University of Birmingham,

Edgbaston,

Birmingham,

UK. Tel - +44 (0)121 414 2896

Email - s.thomas@bham.ac.uk

# Supplementary figure legends

**Supplementary figure 1. Generation of and Fhod1 transgenic mice.** A) The gene trap vector was inserted between exons 1 and 2 of the *Mus musculus* Fhod1 gene. B) Genotyping strategy for Fhod1 KO mice. Primer pair 1 and 2 would produce a ~500bp product in WT mice and primer pair 2 and 3 would produce a ~300bp product in Fhod1 KO mice. C) Detailed map of the gene trap vector.

**Supplementary figure 2. Genotyping and phenotyping of knockout mice**. A) Example PCR products for mDia1 and Fhod1 transgenic mice. mDia1 wild type band = 320bp; mDia1 knockout band = 450bp. Fhod1 wild type band = 500bp; Fhod1 knockout band = 300bp. B) Analysis of phenotypes for the mdia1 KO, Fhod1 KO and DKO mice demonstrating that inheritance for this mice is Mendelian. C) Full western blots for mdia1, Fhod1 and Daam1 expression in mouse platelets.

**Supplementary figure 3. Analysis of white blood cells from transgenic mouse lines.** A) Total white blood cell count in peripheral blood from the four mouse lines. The percentage of white blood cells that are B) lymphocytes, C) neutrophils, D) monocytes, E) basophils and E) eosinophils are shown. Each data point represents 1 mouse. Error bars represent mean ± SD

**Supplementary figure 4. Analysis of resting mouse platelets, platelet clearance and platelet recovery.** A) Resting platelet surface GPIbα levels. Bars represent mean ± SEM (n=10). B) Platelet lifespan and C) platelet recovery for all four genotypes. Bars represent mean ± SEM. For B) & C), each point represents data from between 3 and 6 mice.

**Supplementary figure 5. Analysis of megakaryocyte development.** A) Representative image of a section of femur stained and counted for megakaryocytes. The top image shows a tile scan of the whole bone sample and the lower image a zoomed in region showing megakaryocytes (yellow arrows). B) Proportion of cells in freshly flushed bone marrow that are megakaryocytes (n=4) error bars represent mean ± SD. C) Gating strategy for Ploidy measurements. D) Representative flow cytometry traces for ploidy measurements of freshly isolated megakaryocytes. E) Dot plot for the DNA ploidy measurements from Figure 2B. F) DNA ploidy profile for megakaryocytes cultured *in vitro* showing loss of differentiation delay in mDia1 and DKO cells (n=4).

**Supplementary figure 6. Megakaryocyte proplatelet formation and spreading assays**. A) Representative overview of proplatelet formation assay showing a tiled 6000 μm x 6000 μm field of view. Cells identified by the segmentation workflow are overlaid with coloured masks. B) Example zoom in of a small region of cells showing the just the segmented mask. A number of cell phenotypes can be observed in the image. Small cell fragments or debris identified by the analysis (e.g. bottom right) are filtered out during subsequent processing. C) Example images showing the classes used for automated classification of segmented cells. The left column shows the fluorescence image and the right column shows this overlaid with the segmentation mask. In the unspread class, an example of both an unspread megakaryocyte and undifferentiated cell can be seen. D) Representative images of cultured bone marrow megakaryocytes on fibrinogen for 3 hrs and stained for actin and tubulin. Scale bar = 25 µm.

**Supplementary figure 7. Analysis of platelet spreading.** Representative image of platelets spread on A) fibrinogen, b) fibrinogen + thrombin and C) collagen for 45 min and stained for actin and tubulin. Scale bar = 10 µm.

**Supplementary figure 8. Platelet functional assays.** A) Representative aggregation traces of the four mouse genotypes to collagen or thrombin. B) Representative flow cytometry plots for platelet surface receptors and C) fibrinogen binding and P-selectin expression on platelets stimulated with thrombin. D) Representative end point images for aggregation under flow experiments on collagen at 1000s^-1^. E) Mean percentage increase in fluorescence intensity from *in vitro* flow experiments for each genotype (n=4). Two way ANOVA revealed no significant difference between genotypes (P=0.65).

**Supplementary figure 1**


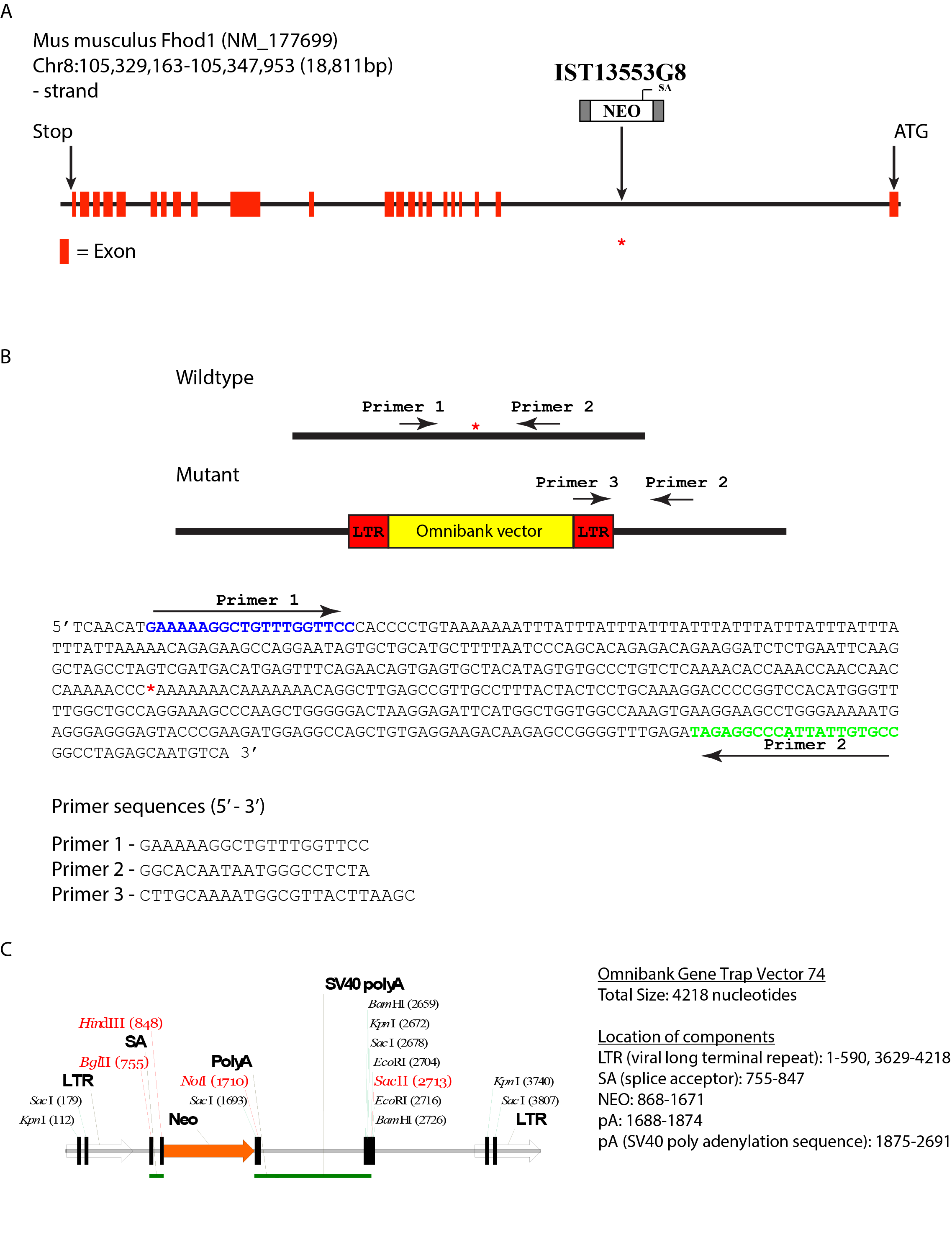


**Supplementary figure 2**


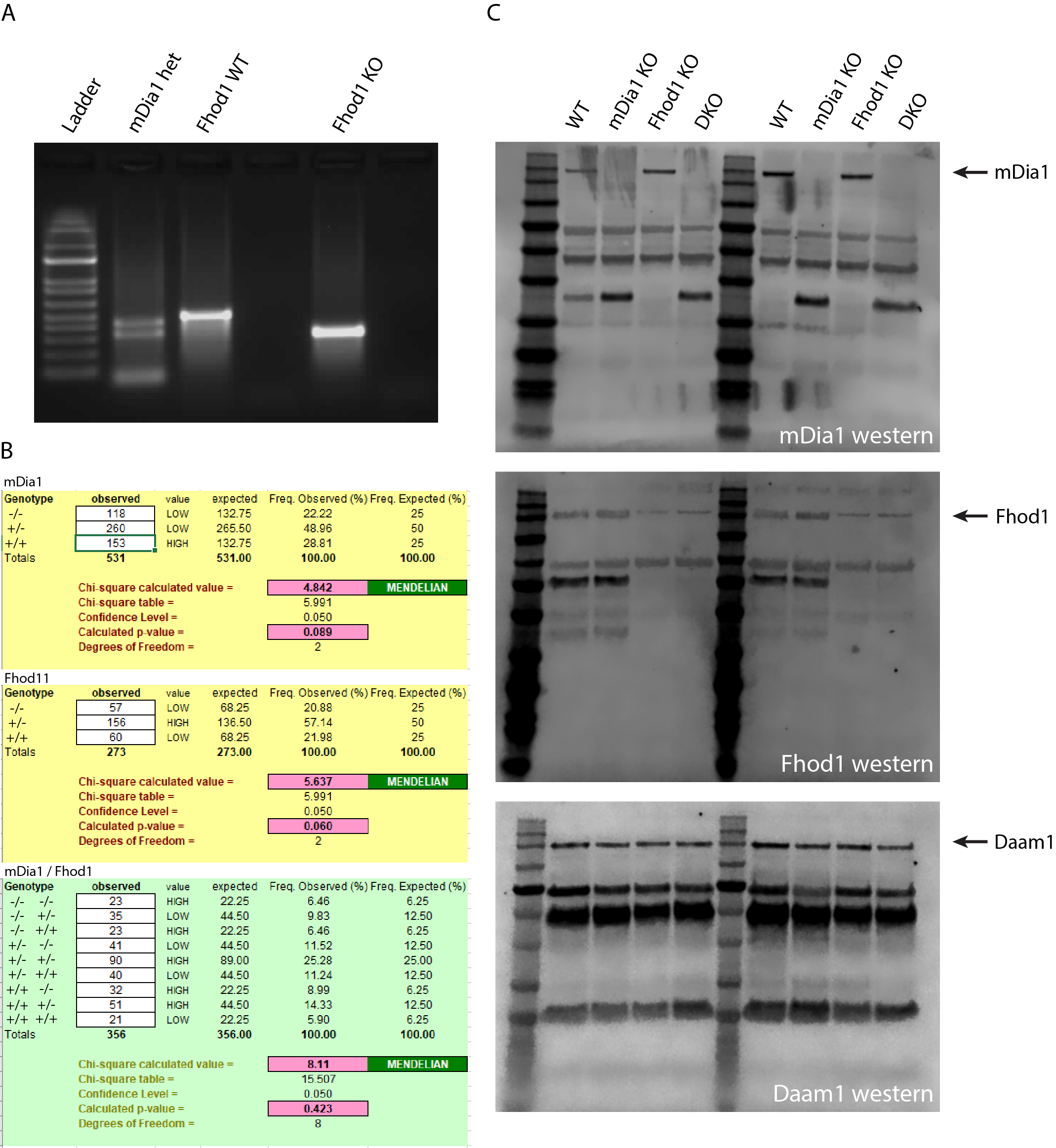


**Supplementary figure 3**

**
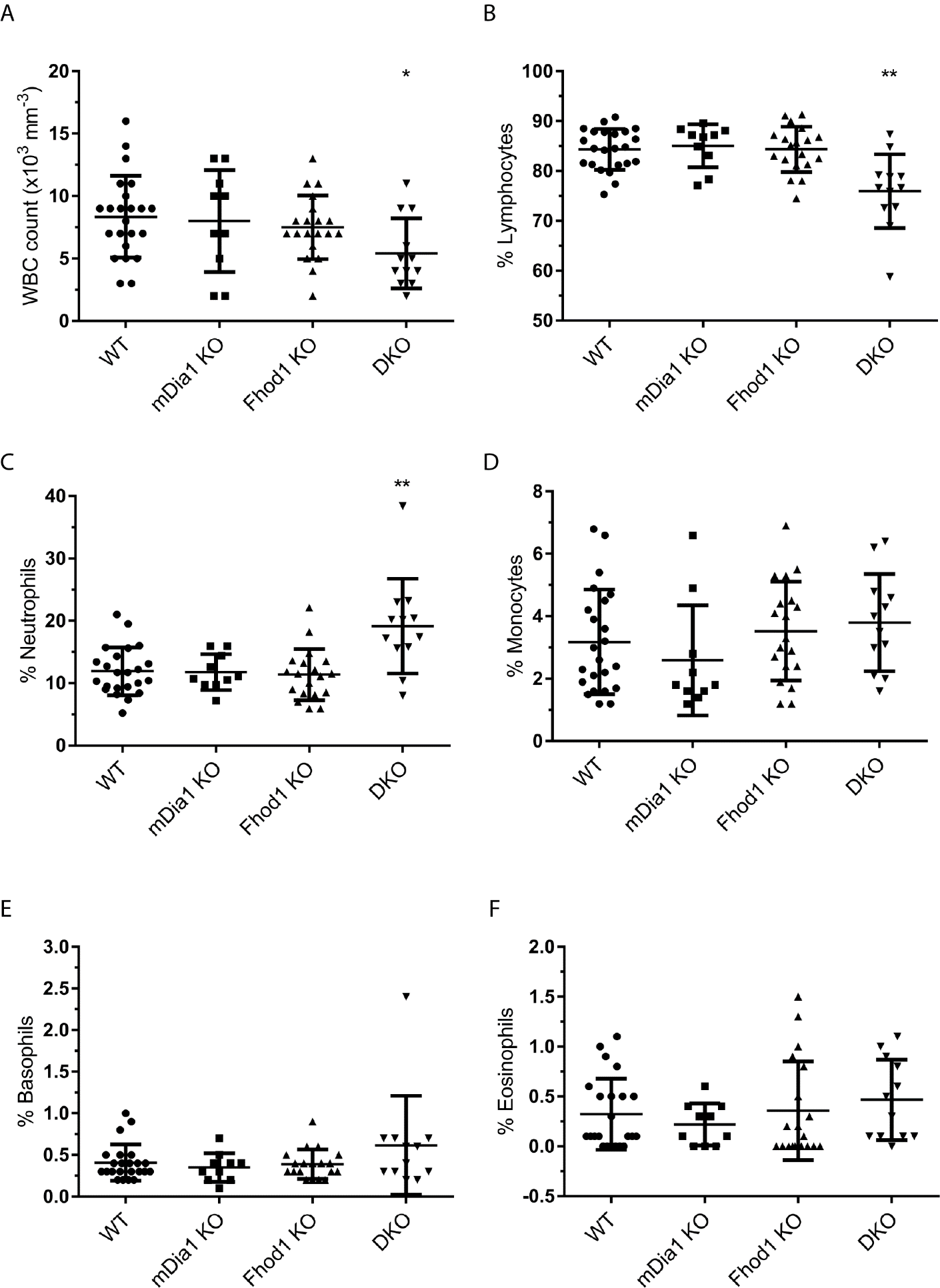
**

**Supplementary figure 4**

**
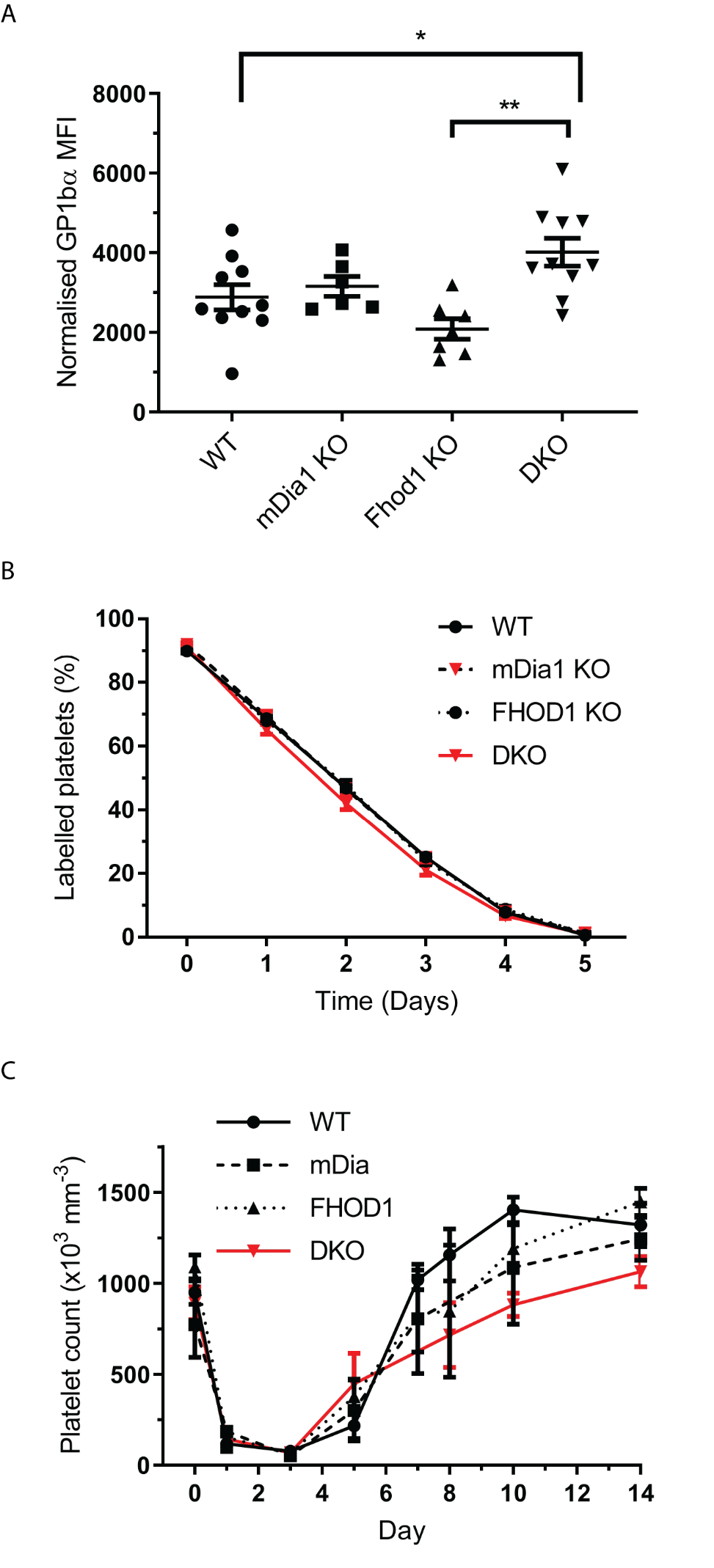
**

**Supplementary figure 5**

**
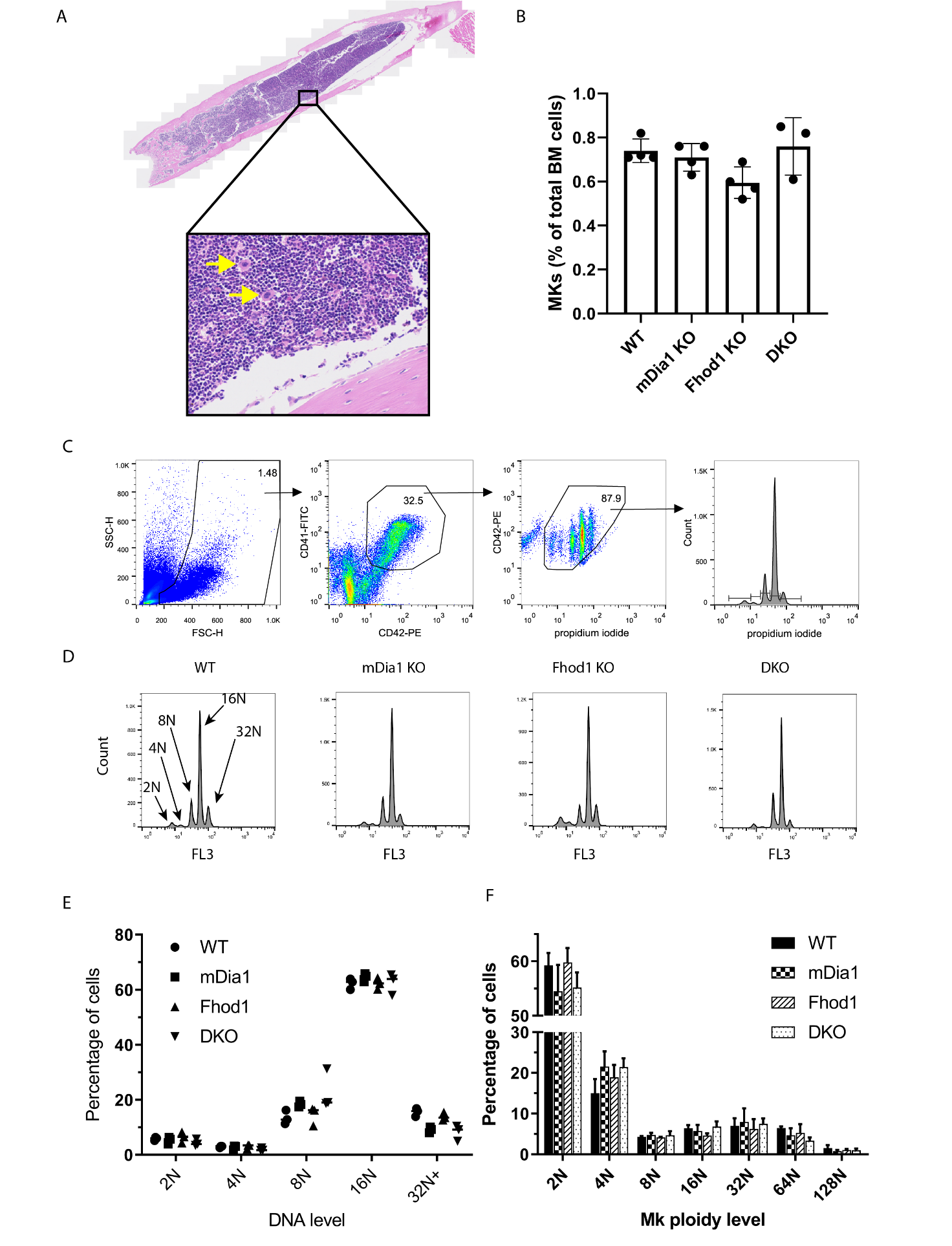
**

**Supplementary figure 6**

**
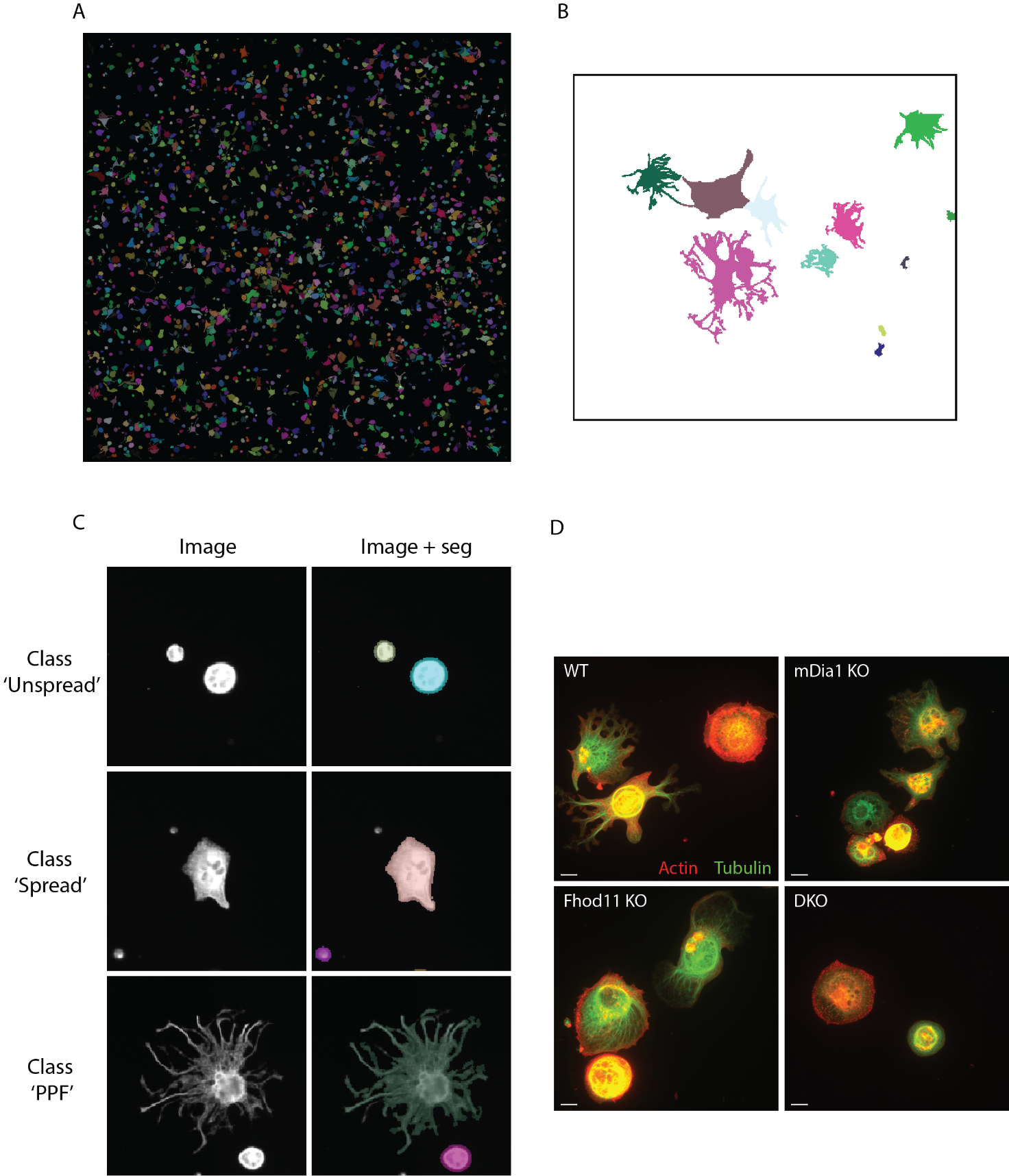
**

**Supplementary figure 7**

**
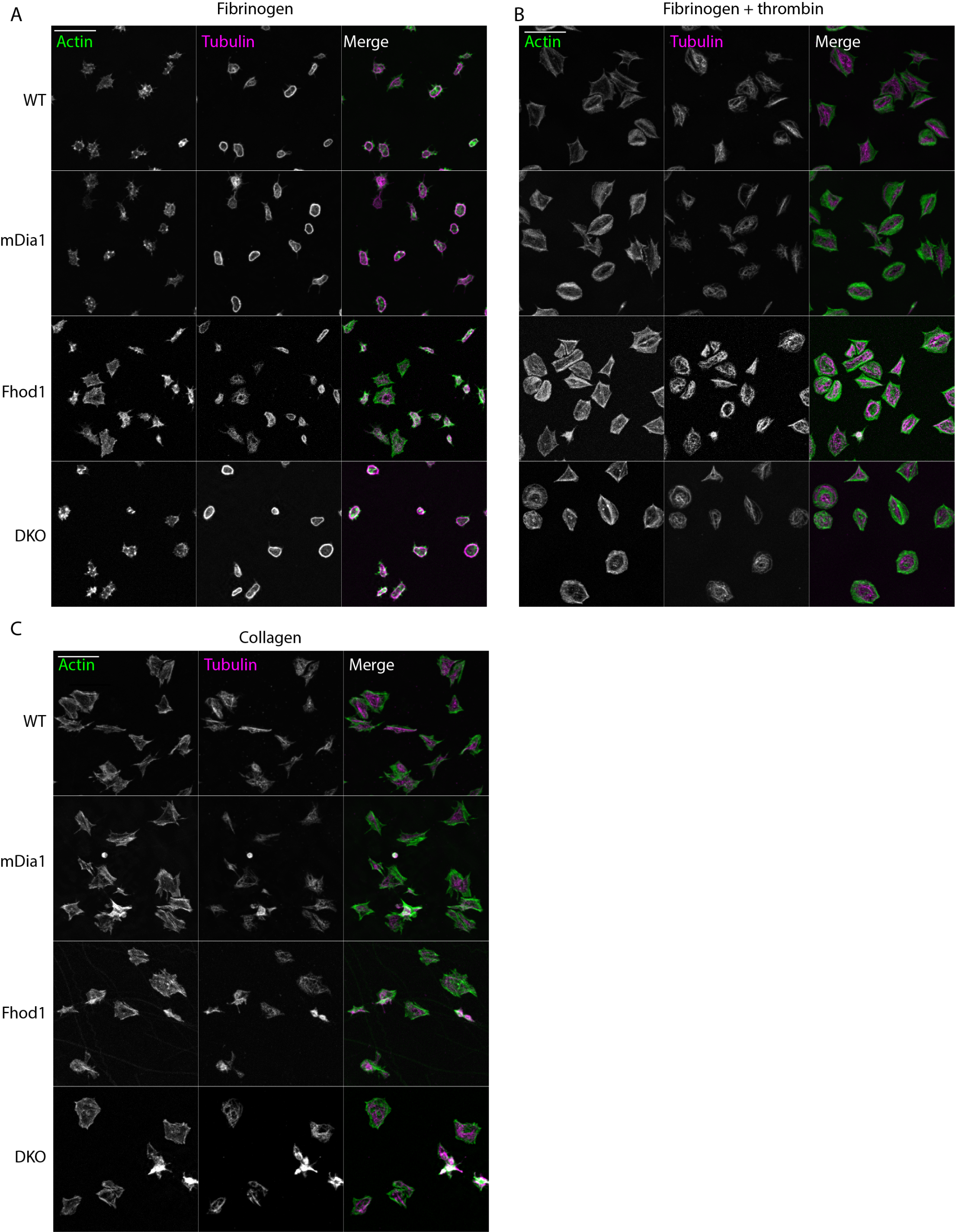
**

**
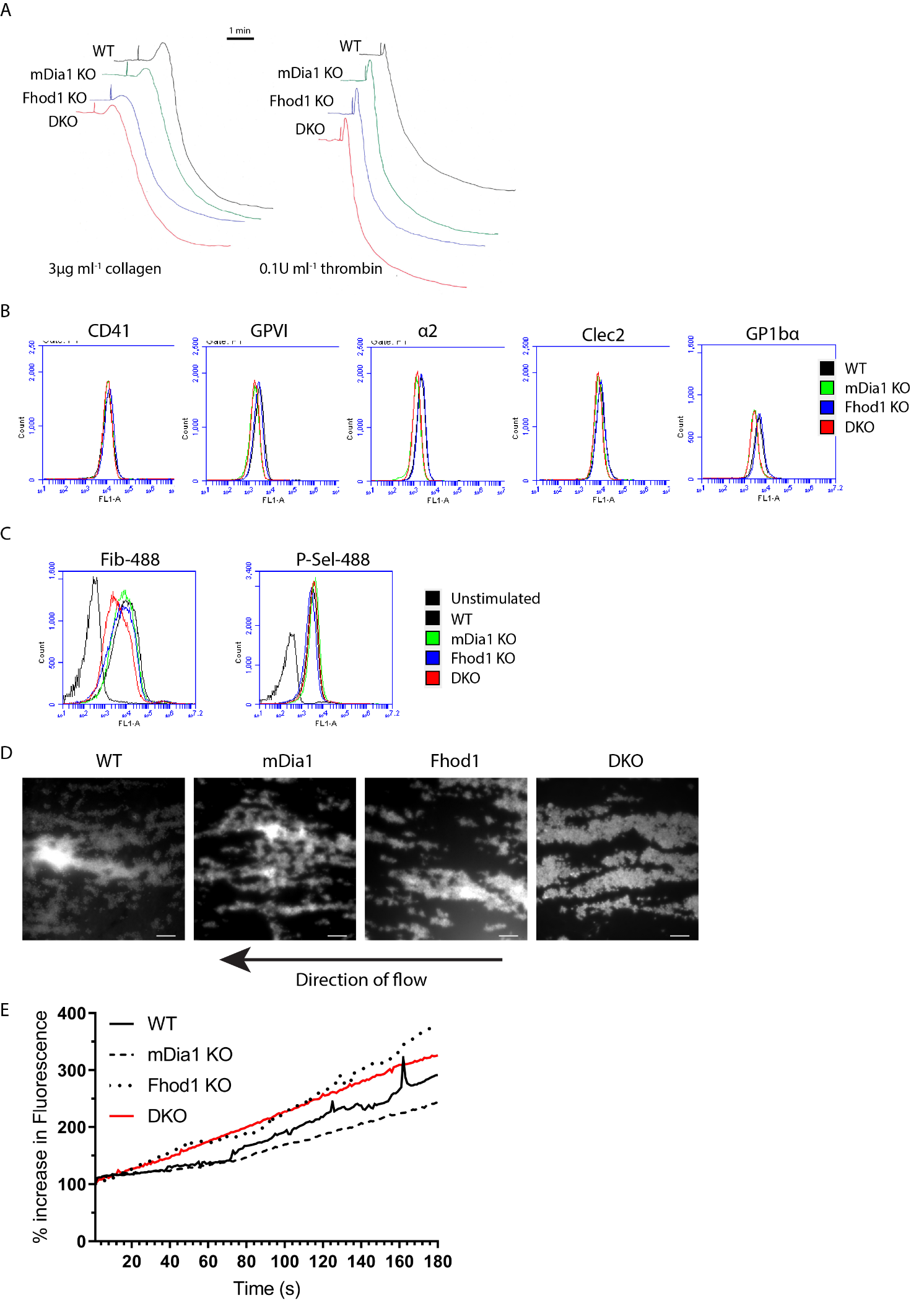
Supplementary figure 8**
